# How do navigation programs address the needs of those living in the community with advanced, life-limiting Illness? A realist evaluation of programs in Canada

**DOI:** 10.1186/s12904-023-01304-3

**Published:** 2023-11-15

**Authors:** Robin Urquhart, Cynthia Kendell, Kathryn Pfaff, Kelli Stajduhar, Linda Patrick, Carren Dujela, Sarah Scruton, Faith Fauteux, Grace Warner

**Affiliations:** 1 Department of Community Health and Epidemiology, Dalhousie University, Room 413, Halifax, NS Canada; 2 Department of Medicine, Dalhousie University, Halifax, NS Canada; 3 Faculty of Nursing, University of Windsor, Windsor, ON Canada; 4 School of Nursing, University of Victoria, Victoria, BC Canada; 5 School of Occupational Therapy, Dalhousie University, Halifax, NS Canada

**Keywords:** Navigation, Palliative care, Realist evaluation

## Abstract

**Background:**

We sought to identify innovative navigation programs across Canadian jurisdictions that target their services to individuals affected by life-limiting illness and their families, and articulate the principal components of these programs that enable them to address the needs of their clients who are living in the community.

**Methods:**

This realist evaluation used a two-phased approach. First, we conducted a horizon scan of innovative community-based navigation programs across Canadian jurisdictions to identify innovative community-based navigation programs that aim to address the needs of community-dwelling individuals affected by life-limiting illness. Second, we conducted semi-structured interviews with key informants from each of the selected programs. Informants included individuals responsible for managing and delivering the program and decision-makers with responsibility and/or oversight of the program. Analyses proceeded in an iterative manner, consistent with realist evaluation methods. This included iteratively developing and refining Context-Mechanism-Outcome (CMO) configurations, and developing the final program theory.

**Results:**

Twenty-seven navigation programs were identified from the horizon scan. Using specific eligibility criteria, 11 programs were selected for subsequent interviews and in-depth examination. Twenty-three participants were interviewed from these programs, which operated in five Canadian provinces. The programs represented a mixture of community (non-profit or volunteer), research-initiated, and health system programs. The final program theory was articulated as: navigation programs can improve client outcomes if they have supported and empowered staff who have the time and flexibility to personalize care to the needs of their clients.

**Conclusions:**

The findings highlight key principles (contexts and mechanisms) that enable navigation programs to develop client relationships, personalize care to client needs, and improve client outcomes. These principles include staff (or volunteer) knowledge and experience to coordinate health and social services, having a point of contact after hours, and providing staff (and volunteers) time and flexibility to develop relationships and respond to individualized client needs. These findings may be used by healthcare organizations – outside of navigation programs – to work towards more person-centred care.

**Supplementary Information:**

The online version contains supplementary material available at 10.1186/s12904-023-01304-3.

## Background

Aging demographics and the increasing prevalence of chronic diseases are intensifying the pressures on healthcare systems to provide person- and family-centred care as people live with advanced, life-limiting illness and plan for end of life (EOL). Current primary healthcare systems are unable to meet the needs of many of these individuals, despite efforts to redesign systems to better meet the needs and goals of patients with serious and life-limiting illnesses [1–4]. Research from across Canada has demonstrated that care provided to those approaching EOL is often highly fragmented and largely hospital-based, [5–7] despite most people preferring to remain and die at home, when possible [8–10]. Many Canadian studies and reports have also revealed a lack of patient, family, and provider knowledge of what programs and supports are available for those with advanced, life-limiting illness, leading to poor care coordination and unsuccessful transitions across health care sectors [11–13]. At the same time, both patients and providers report that the current arrangement of providers, services, and referral pathways in their communities is confusing and difficult to navigate [14–17]. Breakdowns in communication or coordination occur at many junctures – with the consequences being that patient and family needs remain unmet, leading to high levels of distress, frequent high acuity health care use, and poor quality of life [18].

Improved integration of health/social care sectors and providers is key to ensuring better coordination and smoother transitions for people as they approach the EOL, and optimizing outcomes. One approach to improved integration is the provision of navigation programs that can educate patients and families (hereafter referred to as clients), link them to critical health system and community services and supports, and facilitate coordination across care settings. A robust body of evidence demonstrates navigation is effective at improving access to cancer screening and treatments, [19–22] particularly for vulnerable patient groups, and helping community-dwelling older adults reduce hospital readmissions [23, 24]. Several research teams across Canada [25–27] are testing models of health navigation, including volunteer- and nurse-led navigation, to help clients with advanced, life-limiting illness and their families to access services and supports, and improve their quality of life, as they approach EOL. An improved understanding of whether and how navigation programs help clients with advanced, life-limiting illness meet their needs and support their care in the community would help inform practice and policy around this critical health system issue.

Given this context, our study objectives were to [1] identify innovative navigation programs across Canadian provinces and territories that target their services to individuals affected by life-limiting illness and their families, and [2] articulate the principal components of these programs that enable them to address the needs of their clients who are living in the community. Using a realist evaluation framework, the second objective sought to understand what works about these navigation programs, for whom, and in what contexts to improve client and health system outcomes.

## Methods

### Study design

We employed a phased study design to address our objectives. Specifically, we conducted a horizon scan of innovative community-based navigation programs across Canadian jurisdictions (Objective 1) and semi-structured interviews with key informants from the identified programs (Objective 2). Approval to conduct the study was received from the Nova Scotia Health Research Ethics Board, University of Victoria Research Ethics Board, and University of Windsor Research Ethics Board.

### Conceptual approach

We applied realist evaluation [28] as a methodological framework to evaluate navigation programs focused on helping clients access services and supports that enable them to achieve care goals as they approach EOL. Realist evaluation research investigates whether, why, and how complex interventions work – in other words, questions related to what works, for whom, under what circumstances, and to produce which outcomes. Realist evaluation is particularly relevant to investigating the delivery and impact of navigation programs (a complex intervention), given the multifaceted health system in which these programs are delivered, including the multitudes of actors, social processes, and structures. In effect, we aimed to understand the relationships amongst the different clinical and community settings in which navigation is being delivered (the Context) and the elements of the programs themselves (the Mechanisms), and how they lead to impacts (the Outcomes). The final context-mechanism-outcome (CMO) configurations, or *program theories*, are a foundation of realist evaluation.

### Data collection

First, we conducted a horizon scan to identify innovative community-based navigation programs, using an adapted Agency for Healthcare Research & Quality approach [29]. We specifically sought innovative programs at the provincial levels aimed at addressing the needs of community-dwelling individuals affected by life-limiting illness and approaching EOL, and their families. Aligned with the Health Council of Canada’s Innovative Practices Evaluation Framework, [30] the inclusion criteria for innovative programs was:

1. The program addressed a need or gap related to a current health care issue;
2. The program was not a specific drug, surgical, or medical intervention; and.
3. The program was perceived as new and innovative by those for whom the program is intended, those delivering the program, and/or other key stakeholders in the health care system.

Sources of identification included grey literature, websites, and a nomination method. The nomination method involved: sending a Request for Nominations to three or more research, clinical, and policy leaders in palliative care in each province with the selection criteria for innovative programs; compiling an inventory of nominated programs; and contacting the primary contacts of each relevant program to acquire preliminary data on the program, including its aims, components, governance, delivery structure, and evaluation findings. From this process, eligible programs were discussed amongst all team members (including researchers, clinicians, decision-makers, and patients/caregivers). The final selection of programs for the realist evaluation was based on specific criteria (see Box 1), which included (1) meeting the components of navigation programs [31] and (2) aligning with a palliative approach to care [32].

For the realist evaluation, semi-structured interviews were conducted with key informants from each of the selected programs. Key informants included individuals responsible for managing and delivering the program and decision-makers with responsibility and/or oversight of the program. These individuals were identified via the nomination process above and/or publicly available information (e.g., web searches). A research coordinator (CK in Nova Scotia, FF in Ontario, or CD British Columbia) contacted each potential participant via email or phone. Upon a willingness to participate, the research coordinator then initiated the informed consent process and scheduled the interviews.

All interviews were conducted by a research coordinator experienced in qualitative methods [CK, FF, CD]. The interviews focused on program context; how the program sought to improve awareness of, access to, and/or coordination between needed services, and support clients’ EOL care goals; whether the program achieved these aims; and the degree and nature of integration with primary healthcare services and systems of care. The interview guide contained open-ended questions with related probes. See supplementary file. Per a realist approach, interview guides were adapted to explore and expand on salient theoretical constructs as the program theory was being developed. Interview questions were also adapted as needed based on the person being interviewed and his/her role in the program.

### Data analysis

Data from the horizon scan was summarized descriptively in tables for each identified program. This helped team members discuss and select the final programs for inclusion in the realist evaluation. Transcripts from the semi-structured interviews were read and re-read by members of the research team [RU, GW, KS, KP, LP, CK, FF, CD] to familiarize themselves with the dataset and gain a working understanding of the navigation programs and their components, the contexts in which they are delivered, and their potential mechanisms. From these iterative reviews, initial CMO configurations were developed, with team members meeting regularly over the course of one year to add to these CMOs and refine them. Next, one team member [SS] coded each transcript to identify key concepts (context, mechanisms, and outcomes) and refine the overall program theory. Using techniques common to thematic analysis, [33] this process entailed reviewing the coded data, combining similar codes to generate higher-level concepts, and interpreting the meaning of these concepts across the dataset. The analysis continued to be flexible and iterative in nature, with team members meeting regularly to review, question, and refine key concepts. Following the approach of Dalkin, [34] which posits that program *resources* are introduced in a context in a way that enhances or alters an individual’s *reasoning* (reaction or behavior), the analysis differentiated those mechanisms that represented resources and those that represented reasoning. The final program theory relates to those components of innovative community-based navigation programs that help them address the needs of individuals affected by life-limiting illness and their families as they approach EOL.

## Results

Twenty-seven programs were initially identified from the horizon scan. After several team meetings to discuss and review these programs, 11 were selected to move forward to the realist evaluation. Programs were excluded when team members felt the program did not meet the criteria outlined in Box 1.

**Fig. 1:**
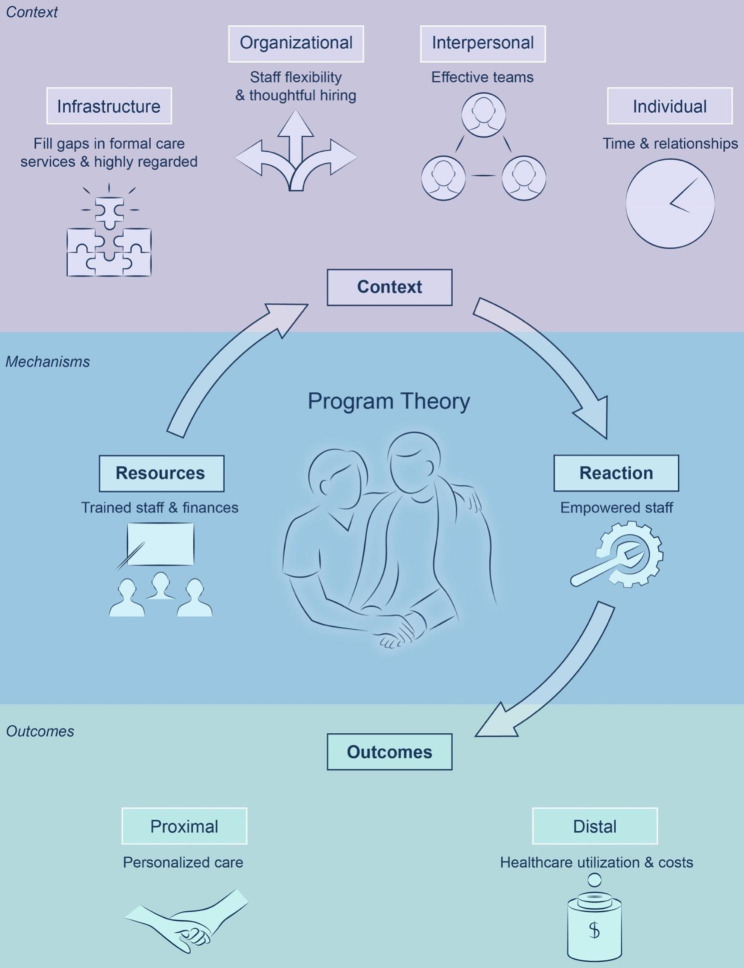
Final program theory and its associated components (context, mechanism, and outcomes). The final program theory relates to those components of innovative community-based navigation programs that help them address the needs of individuals affected by life-limiting illness and their families as they approach end-of-life

Twenty-three participants were interviewed from the selected 11 navigation programs, which operated in five Canadian provinces. They represented a mixture of community (non-profit or volunteer), research-initiated, and health system programs. The final program theory was articulated as: *Navigation programs can improve client outcomes if they have supported and empowered staff who have the time and flexibility to personalize care to the needs of their clients*. Figure 1 depicts this final theory and its associated components (context, mechanism, and outcomes).

### Context

The context at multiple levels is germane to how navigation programs operate and how they improve client outcomes. At the broader *infrastructure (system) level*, participants discussed how navigation programs address gaps in formal care services that are unable to tailor their services to client needs. In fact, many participants felt their programs existed due to gaps in primary care, which would ideally fulfill a navigation function. This was highlighted by the following two participants, who said:

*I think because of the, the coordination that can happen, it really does fill a gap more than anything because…I can give you an example. … As a system navigator, I have many patients where they, they need something but the barrier to them accessing it to me feels very ridiculous. So for example, I work very closely with a palliative patient who is homebound but frequently needs all kinds of stuff. Like they were involved in the internal medicine rapid access clinic and they ordered all these tests and things… Obviously, she doesn’t drive. She barely walks. Her partner can’t get her to these appointments, just even… like I arrange transportation but even getting somebody at the hospital to get her out of the transportation and you know the 20 feet to the ____clinic was a challenge and there was basically this appointment was just not going to happen because there was nobody to be able to do it and because I, you know, because I exist, this position exists, I’m able to say, ‘you know what, I’ll meet transport there and I’ll do it and I’ll facilitate access’ because this patient needs to have this special lens. They need to have these tests done and if nobody else can go then I’m gonna go. But what a huge gap, right? To say, okay, okay you know you have to be able to walk into the building or and then the only other option is what she would normally do and that would be to call an ambulance because she, you know, that’s the way to get to the hospital. The cheapest [way] is to call an ambulance and that was her plan. She was like ‘well I’ll just call an ambulance. They’ll take me.’ And I’m like ‘well no, that’s not a good use of healthcare dollars either.’ So I think that [this program] has the potential to fill some of the gaps that exist.* [Participant 5]

*In some ways, we’re filling that gap, you know, that gap right now in particular as our human resource group, primary care, is challenged. We certainly fill that gap where patients don’t have to fall through a gap.* [Participant 7]

Participants also described strong relationships and high levels of trust with organizations who complement their services. This included community-based organizations, home and continuing care, paramedicine, social work, and specialist (e.g., organ failure) clinics. As stated by one participant:

*So, the other people that we link with, they’re not officially part of our team but we really engage our stakeholders. We engage the special patients program at [Emergency Health Services], we engage our partners at continuing care, um, we engage with the rehab clinic. So I think the other thing that [we do] is to say ‘What are our community resources that can help our patients be cared for?’* [Participant 7].

In fact, participants described how their programs were highly regarded across community and health sectors. For example, many community-based navigation organizations described having exceptional integration with individuals and programs in the formal healthcare system. As a result, program staff were aware of available services and supports and able to refer their clients who needed these in a timely manner. Moreover, the high regard they experienced allowed them to find solutions alongside other community services. As one participant said:

*The volunteers become quite expert in knowing what some of the organic resources are in their community. Who delivers groceries, who has driving services, who has pastoral visiting programs, what are some of the unique programs we have in this community? And then as they work with their client over time, they connect them to those services. So, in one rural community, for example, a woman in a wheelchair needed to see a lawyer but, believe it or not, there was not a single lawyer in that community that had wheelchair access. So, [the program] worked with the lawyers in the community to do a home visit, to make sure they realized they weren’t wheelchair accessible. So, just really practical kinds of things that make all the different to health care, but health care people don’t typically have the time or the energy to resolve.* [Participant 3]

Despite strong relationships with health and community-based services, participants from more than half of the navigation programs expressed suboptimal relationships with primary care, and felt that stronger relationships with primary care would help them better serve their clients.

*They’re getting better but it’s still not the way it should be. We should be a referral, like chiropractic or massage or blood work to be honest with you. That’s our goal, that’s our mission … to actually get prescription pads in every primary care office, every doctor’s office, so that they know there’s a support system and it’s just a matter of doing the referral and getting it sent to us and us contacting those [clients].* [Participant 1]

At the *organizational* level, participants discussed three key elements that ultimately enable them to meet their clients’ needs. First, staff were given the flexibility to personalize care so that they address individualized client concerns and preferences. These organizations recognized that there is no one-size-fits-all solution for addressing the needs of persons with life-limiting illness, and that staff need to adapt and tailor their practices as required. As described by one participant:

*I think that’s another huge benefit of the program, is that we work a lot in the grey. There’s no black and white in our life. Because if we did it that way, it just wouldn’t work. So it’s very flexible. It’s, like I said, back to the patient-centred type of care that … You know, what works for one person doesn’t work for another. And sometimes we need to bend and flex and change the rules a little bit, or make up our own rule this way, or whatever, in order to help things move along.”* [Participant 8].

One example of this flexibility is home visits. Participants described how visiting clients in their homes are an important part of several programs, preventing unnecessary and inconvenient travel on the part of the client and allows staff to ensure a client’s home is set up to safely and effectively support them. One participant described it like this:

*Clinicians have also said that it’s very helpful to have volunteers be the eyes and ears of the health care team in the person’s home. So … in the context of people who were just recently discharged from hospital, we made it a priority that the volunteer … would need to go back into the home within two days of the person being discharged. There was a lot of discussion about what’s the right time, so we need someone to have enough time to settle and get back home but we also want to make sure that the supports that the person needs when they transition from home from hospital are there in a timely way, and that having the eyes and ears in the home can sometimes support and help prevent them from going back to hospital right?* [Participant 4]

Second, participants emphasized the careful and thoughtful approach of their organizations upon hiring staff and recruiting volunteers to ensure the program encompasses people with the right “fit” in terms of skillsets, knowledge of the local context (i.e., additional services and supports that could be leveraged to support clients), and personal values that are aligned with those of the program. In fact, many expressed that hiring and recruitment practices were essential to developing teams that are able to collaboratively and effectively work together for the benefit of clients. As one participant said when asked about their program’s success:

*Just the team that we have. There’s a very strict selection process for the people who join the team. It’s certainly not for everybody. It’s a really different way of practicing. And, so I think that really helps to facilitate the success of the program.* [Participant 8]

Participants further described the selection of staff and volunteers as being highly tailored to the needs of the program and its clients. For example, participants from some health system-based navigation programs discussed only hiring staff with prior experience working in the community and knowledge of community-based health and social services. Similarly, participants described how several of the navigation programs are connected to, and leverage volunteers from, established hospice programs and organizations that have well-developed policies, procedures, and training programs. Many of these volunteers are selected because they are retired healthcare professionals who have experience with similar client populations.

Third, organizations provide clients direct access to supports by ensuring they have a contact person so that clients know they are able to access this person and get a timely response/action. One participant described it like this:

*Well, I think the knowing … As I said, we always say you are not alone. So, most people typically have a Monday to Friday, 8 to 4 job. And we try and stay connected, which means we use our social media. So, if there’s a message that comes on social media, and it’s something that needs addressing right away and can’t wait, then of course we do that. So, it could be 7:00 on a Friday night, and we’re responding to emails or to Facebook messages. Even when I was away on vacation, I was still triaging my emails. So, I think to know that they’re not alone, to know that we’re there to help. I think knowing that if there’s questions that need answers, that we will help them find them. I think just the kindness, the compassion that comes out within the staff as well. The open-door policy. There’s a lot that I think really makes this program what it is.”* [Participant 10].

At the *interpersonal* level, participants described their programs as comprised of effective teams of problem solvers who have complementary skills that allow them to meet client needs. As one participant stated, they take an “all hands-on-deck approach” [Participant 10] to meeting their client’s needs. Many discussed how their different perspectives and roles allow them to think through and create personalized solutions to client issues. As said by one participant:

*That was an interesting, eye-opening learning for me because the [team] huddle sort of forces the team to come together in a way, and look at one patient at a time, and to engage in an interprofessional discussion about that one person. And I think that the patient benefits from having multiple lenses looking at, you know, some of the issues that they’re bringing up in their report.”* [Participant 4].

Participants also discussed how they often operate as ‘teams of problem solvers’ across organizations, given their strong relationships.

*We are a social service agency … [yet] we’re able to collaborate with hospital staff very easily because we have the connections, we know who to call. So definitely, in that case, that helps the program a great deal. We do have services. We have personal support services, housekeeping, that sort of thing. So, we were able to leverage some of these resources to help people in the community through this program.* [Participant 17]

At the *individual* level, participants discussed how staff value taking the time necessary to build relationships with clients and advocate for client and family needs. In fact, they discussed how their programs allow them to be relationship-focused versus task-focused, enabling them to know their clients in a more holistic way. As described by one participant:

*Home visits, that’s probably one of my, that’s probably my favourite part of my job. So every opportunity I get, I really do take it. … By me being there and being able to chat to them, talk about the pros and the cons, or completely go off subject and talk about family while [colleague is] setting up the equipment, it really seems to eliminate some of that stress and anxiety that they have. So that is one of our key parts of my position … I’m going to come over for a visit and stop for coffee, and I’m going to drop off some stuff for you. So, it really is a key component to our program. If I’m able to take one thing off their plate, whether it be make a phone call or just come down and visit, anything to make their lives easier, their quality of life easier, whether it be for our client or our family, that’s kind of where our mindset is. … So the relationship really evolves. And I find over time, the relationship, it no longer becomes a [service provider-client] kind of relationship. They really let us into their hearts, their homes at the most difficult time, and teach us so much about life ourselves.* [Participant 10]

Finally, participants from all programs expressed pride in, and fulfillment from, their programs and what they are able to accomplish. One participant expressed it this way:

*I can say this is one of my favourite jobs that I have ever had. You’d never hear me groaning getting out of bed in the morning, going, ‘oh my goodness, I have to go in to work.’ … There’s things that I’ve taken from each and every one of our clients that I’ve somehow implemented into my own personal life. And one of the reasons for this job was if I could make a difference in just one person’s life, then I’ve done my job. So that’s kind of my question of the day when I’m getting ready to go home, saying, ‘okay, did I make an impact, did I take just one thing off of somebody’s plate today, and was I successful doing so?’ And if I answer yes to that question, I’m like, okay, then I’ve done my job. So I always check myself for that towards the end of the day, and say, ‘okay, what did I do today that really made that difference?’ And I haven’t had a day where I’ve had to go home and say, no, I did not do that.”* [Participant 10].

This job satisfaction often kept the motivated and energized as they worked within a complex system, and often with limited resources, to support the varied needs of clients with life-limiting illness.

### Mechanisms (resources and reactions)

Within this context, well trained staff and volunteers, and sufficient financial resources, were deemed necessary to deliver services as well as to evaluate their impact on clients. In this study, most programs spent considerable time training paid staff and volunteers. Participants from programs that were connected to hospice organizations described how many of their volunteers have already undergone training on how to work with/care for people who are nearing EOL, and are given additional training on navigation skills. High quality training was perceived as a key component toward creating an environment wherein staff and volunteers have the confidence and ability to help clients manage their multitude of needs as they near the end of life.

*Volunteers that are connected with hospice, they go through a huge training. So they have, they have a lot of knowledge. … I think this is the big difference, like a hospice volunteer is aware of all the needs. Like, spiritual care needs, like social needs, those needs that no one else looks at, like they will pay attention. The health care provider will pay attention to the pain, will pay attention to whatever is happening, and not about these other parts that our volunteers could probably be a good helper in identifying and in connecting with the resources in the community. So this is the main thing.”* [Participant 2].

The funding structure for programs differed widely, including government, grant, and donor funding models (and sometimes a combination of these). Nonetheless, most (8/11) programs described limited and unsustainable funding, impeding their ability to deliver services to their client base and sometimes creating an uncertain environment for staff and volunteers. As one participant said:

*We have not continued to have incremental funding. So, I think what it has required us to do is to actually … with every person who’s left, the team has to really carefully evaluate in terms of do they need that position where it is, is there a need, is there a greater need somewhere else? Do we actually need that person or do we need somebody else? So, I think they have been very good at looking at that. But we still have areas of the province where they have small programs or would like to see a further expansion where we’ve not been able to carve out funding to actually enable that. So it is an ongoing dance in terms of looking at, you know, your budget.”* [Participant 9].

As a result of the context and resources provided, participants described staff who are empowered to be person-centred in their approach and to tailor care to clients’ needs. In fact, participants described staff as feeling largely supported in their roles, as described by one participant:

*[This job] can be physically demanding, both emotionally and mentally. … Like I said, the Board and [my supervisor] have been very, very understanding with that, and I think they notice the need. So whenever a staff member is coming in and they’re feeling a little overwhelmed or they need to talk or need a personal day, sometimes they’ll just come in and go, ‘Don’t come to work on Friday. Take a long weekend and enjoy it, and we’ll cover everything for you.’ So that really makes my job easier. And I think the flexibility of it too. … Again, I think that goes hand-in-hand with the organization: the compassion that we show for our staff as well as for our clients and their families. It’s a wonderful organization to work for.* [Participant 1]

These supportive settings enable staff to take the time necessary to build relationships with clients, provide emotional support, and advocate for access to needed medical and community services, equipment, and other supports.

### Outcomes

Participants from all programs described tangible client outcomes from the services they provide. Namely, all participants discussed how their program improves access to the information, equipment, and services that their clients need as they live with life-limiting illness. As one participant stated, “*bringing the services to patients in their home greatly impacts their access to the healthcare*” [Participant 8] Another described their program impacts like this:

*So, I would say that the participants really liked the program. They benefited in a number of different ways, they were saying like, aside from the fact that it did result in some positive health outcomes, you know more physical activity less hospitalizations etc. From a qualitative [point of view], the clients felt that it met needs like, you know, the need for social support, it met the needs like developing a stronger link to their health care team, it met a need around timely services.* [Participant 4]

Participants also discussed increased client activation and personalized care, fewer unmet emotional needs, and prevention of health crises. Participants from four programs, which had conducted formal evaluations, discussed how the program decreased health service utilization, including emergency department visits and hospitalizations. Three of these programs had also demonstrated a reduction of costs due to lower acute care utilization.

*[We] were having a positive impact because we did do surveys and they did do, um, have data on emergency room visits decreasing, and improvements in the number of patients who had advance care planning completion, and patient satisfaction surveys. … [A later evaluation showed that] by enhancing patients’ confidence to manage their illness in their homes and communities, there was sustained and substantial reduction in hospital-based care, shown to prevent emergency visits, reduce hospitalizations and length of stays. And it was also shown to reduce reliance on acute hospital admissions for end of life care.* [Participant 9]

## Discussion

Using a realist evaluation approach, this study sought to understand how innovative community-based navigation programs help address the needs of individuals affected by life-limiting illness and their families as they approach EOL. Specifically, we found that navigation programs improve client outcomes when they support and empower staff to take the time and flexibility to personalize care to clients’ medical and social needs. Our findings further point to the importance of inter-organizational relationships and trust, nimble work environments, thoughtful hiring practices, and training to ensure that staff are indeed equipped to work as teams of problem solvers, and to build relationships with and advocate for their clients. Of note, our findings also revealed that the selected navigation programs were initiated, and continue to exist, due to gaps in the way health services are currently delivered to people with life-limiting illness who live in the community.

A realist review of new palliative care services in England found that highly skilled, client-focused providers, who had access to community resources and were given sufficient time with clients, engendered staff and client confidence and potentially contributed to beneficial outcomes, such as fewer hospital admissions and greater deaths at home [35]. This, and other studies, also found that having a single point of contact that can be reached after hours is critical to meeting the needs of people with life-limiting illness [35, 36]. Focused on navigation programs, our study found that similar contexts and mechanisms resulted in empowered staff and improved client outcomes as they neared EOL. That these findings applied across program settings (e.g., health system, community, and research-initiated) demonstrate there are core principles that make these programs work as opposed to certain organizational structures or characteristics.

With few exceptions, most navigation programs in this study described weak relationships with primary care – despite being well connected to other medical and social programs and services. In fact, many of the navigation programs were initiated due to perceived gaps in the primary care system’s ability to care for people with advanced, life-limiting illness who are living in the community. This is perhaps unsurprising given the increasing patient volumes, persistent shortage of primary care providers, and lack of integration between primary care, acute care, and community services in Canada [37–39]. Conceptually, primary care systems aim to help patients navigate and coordinate their care journey. This role is particularly relevant for older individuals given the longstanding relationships that exist between providers and patients, and providers’ knowledge of patients’ medical history as well as social contexts [40]. However, primary care is poorly positioned to fill this coordination role in many countries, due to both structural and funding barriers [41, 42]. Despite increasing support for interprofessional primary care teams (e.g., Family Health Teams in Ontario), care continues to be disjointed in many parts of the country and ineffective at meeting the multi-faceted needs of persons with advanced chronic disease and/or multi-morbidity [41, 43]. As such, navigation programs will continue to provide critical services and supports for people with advanced, life-limiting illness. Ensuring these programs are adequately resourced will be a key ingredient to realizing and sustaining their beneficial outcomes.

There are several practice implications of our findings. First, although navigation programs may benefit from having staff (or volunteers) with certain clinical experience and/or knowledge, it is similarly important that staff have formal or informal knowledge of available health and social services to ensure clients’ multi-faceted needs are met. Interestingly, primary care providers in Canada have expressed frustration with the complex nature of navigating community-based health and social services, while health and social service providers report struggles communicating with primary care [44]. s, having a point of contact that can be reached after hours is critical for clients who are living in the community with life-limiting illness. Similarly, home visits allow staff to not only minimize unnecessary travel for clients, but also allow them to more effectively assess and address the multiple health and social needs of clients as they near EOL. Finally, it is clear that delivering truly person-centred care requires that staff have flexibility and time to develop relationships with clients and respond to their individualized needs. From a client perspective, accessible, high-quality care means that one receives the right care in the right place at the right time [45]; in other words, one receives personalized care. Unfortunately, traditional professional boundaries, and regulatory and financing models, mean that many health providers operate within relatively inflexible environments [46, 47]. Any real shift to person-centred care will require that health systems embrace the principles identified in this study, and others, to optimize care for those with advanced, life-limiting illness.

This study employed realist evaluation methods to explore the principal components of how navigation programs are able to address the needs of community-dwelling persons with advanced, life-limiting illness. Importantly, the navigation programs selected for study were from five Canadian provinces (representing different health systems in terms of resources and constraints) and situated across varying contexts (representing community, research-initiated, and health system programs). Therefore, the resultant theory should be applicable across many health care contexts. Nevertheless, there are several limitations. First, data collection for the realist evaluation commenced at the start of the global COVID-19 pandemic. This led to challenges in recruiting individuals from the selected programs, given the additional strain the pandemic placed on these programs and their clients. As a result, we did not interview as many individuals as we had initially anticipated. Second, we had initially proposed to interview clients as part of our realist evaluation. However, recruiting clients proved exceptionally challenging as programs did not have the capacity to assist with recruitment during the pandemic. Still, patients and family caregivers were members of the research team and provided their insights into navigation programs and how they work for individuals with life-limiting illness.

## Conclusions

The findings from our realist evaluation suggest that navigation programs are able to improve outcomes for clients when their staff are able to be flexible in their approach and take the time necessary to develop client relationships and personalize care to clients’ needs. The findings also point to key principles (contexts and mechanisms) that enable staff to work in this regard. These principles include selecting staff and volunteers with the knowledge and/or experience necessary to coordinate health and social services, and providing an after-hours point of contact and home visits to enable clients to stay in their communities. These findings may be used by healthcare organizations – outside of navigation programs – to work towards more person-centred care. Of note, staff who worked within these programs felt pride and accomplishment in their work, and their job satisfaction often kept them motivated and energized as they worked to improve client care. The findings, therefore, may also help address issues related to health workforce moral and burnout, which have been heightened worldwide during the global COVID-19 pandemic.

**Table:** 

**Box 1. Eligibility criteria for navigation programs included in the realist evaluation**
1. The program serves community dwelling adults with chronic, life-limiting illness.
2. The program may be situated within the community or within a hospital setting.
3. Within the program there are dedicated individuals who provide navigation services. These individuals may be referred to as “Navigators” (though not necessarily) and may be paid staff or volunteers. Navigation services may include, but are not limited to: patient education, care planning, home visits, fostering of coordination and continuity across health settings, and early identification of and response to health changes [31].
4. The program is a formal program. That is, it must be affiliated with a host organization and have some sort of governance structure and accountability.
5. The program offers services that encompass a palliative approach to care. According to Touzal and Shadd “a palliative approach exists when care simultaneously addresses whole-person needs, enhances quality of life, and acknowledges mortality. This model is applicable to care provided in any setting, by any provider, to any patient with a life-threatening illness, at any point in the illness trajectory” [32].

### Electronic supplementary material

Below is the link to the electronic supplementary material.

Supplementary Material 1

## Data Availability

The datasets generated and/or analysed during the current study are not publicly available due given the qualitative nature of these data, but are available from the corresponding author on reasonable request.

## Abbreviations

CMO: context-mechanism-outcome
EOL: end of life

## References

[1] The British Columbia Patient-Centred Care Framework. Available at: http://www.health.gov.bc.ca/library/publications/year/2015_a/pt-centred-care-framework.pdf., 2015.

[2] Duncan D. Patient First Review. Saskatchewan Health Initiatives. Available at: https://www.saskatchewan.ca/government/health-care-administration-and-provider-resources/saskatchewan-health-initiatives/patient-first-review.

[3] Alberta Health Services. Patient First Strategy. Available at: https://www.albertahealthservices.ca/info/Page11981.aspx.

[4] Ministry of Health and Long-Term Care. Patients First: Action Plan for Health Care Message from the Minister of Health and Long-Term Care. Available from: http://www.ontario.ca/health. 2015.

[5] Heyland DK, Lavery JV, Tranmer JE (2000). Dying in Canada: is it an institutionalized, technologically supported experience?. J Palliat Care.

[6] Canadian Institute for Health Information. Health Care Use at the End of Life in Western Canada. Available at: http://secure.cihi.ca/cihiweb/products/end_of_life_report_aug07_e.pdf. Ottawa, ON: Canadian Institute for Health Information, 2007.

[7] Fowler R, Hammer M (2013). End-of-life care in Canada. Clin Invest Med.

[8] Higginson IJ, Sen-Gupta GJ (2000). Place of care in advanced cancer: a qualitative systematic literature review of patient preferences. J Palliat Med.

[9] Gomes B, Calanzani N, Gysels M (2013). Heterogeneity and changes in preferences for dying at home: a systematic review. BMC Palliat Care.

[10] Burge F, Lawson B, Johnston G (2015). Preferred and actual location of death: what factors enable a Preferred Home Death?. J Palliat Med.

[11] Morrison RS (2017). A national palliative care strategy for Canada. J Palliat Med.

[12] Saint Elizabeth (2013). Highlights from the Environics End-of-Life Care Survey for Saint Elizabeth.

[13] Burge F, Lawson B, Johnston G (2014). Bereaved family member perceptions of patient-focused family-centred care during the last 30 days of life using a mortality follow-back survey: does location matter?. BMC Palliat Care.

[14] Marshall D, Howell D, Brazil K (2008). Enhancing family physician capacity to deliver quality palliative home care: an end-of-life, shared-care model. Can Fam Physician.

[15] Seow H, Brazil K, Sussman J (2014). Impact of community based, specialist palliative care teams on hospitalisations and emergency department visits late in life and hospital deaths: a pooled analysis. BMJ.

[16] Katz A, Martens P, Chateau D (2014). Do primary care physicians coordinate ambulatory care for chronic Disease patients in Canada?. BMC Fam Pract.

[17] Aubin M, Giguere A, Martin M (2012). Interventions to improve continuity of care in the follow-up of patients with cancer. Cochrane Database Syst Rev.

[18] Burton R. Health policy brief: care transitions. Health Aff (Millwood; 2012.

[19] Percac-Lima S, Ashburner JM, Rigotti NA (2018). Patient navigation for Lung cancer screening among current smokers in community health centers a randomized controlled trial. Cancer Med.

[20] Rodday AM, Parsons SK, Snyder F (2015). Impact of patient navigation in eliminating economic disparities in cancer care. Cancer.

[21] Freund KM, Battaglia TA, Calhoun E (2014). Impact of patient navigation on timely cancer care: the Patient Navigation Research Program. J Natl Cancer Inst.

[22] Ko NY, Darnell JS, Calhoun E (2014). Can patient navigation improve receipt of recommended Breast cancer care? Evidence from the National Patient Navigation Research Program. J Clin Oncol.

[23] Balaban RB, Galbraith AA, Burns ME (2015). A patient Navigator intervention to Reduce Hospital readmissions among high-risk safety-net patients: a Randomized Controlled Trial. J Gen Intern Med.

[24] Di Palo KE, Patel K, Assafin M (2017). Implementation of a patient Navigator Program to reduce 30-day Heart Failure readmission rate. Prog Cardiovasc Dis.

[25] Dolovich L, Oliver D, Lamarche L (2016). A protocol for a pragmatic randomized controlled trial using the Health teams advancing patient experience: strengthening Quality (Health TAPESTRY) platform approach to promote person-focused primary healthcare for older adults. Implement Sci.

[26] Pesut B, Duggleby W, Warner G (2017). Volunteer navigation partnerships: piloting a compassionate community approach to early palliative care. BMC Palliat Care.

[27] Pesut B, Hooper B, Jacobsen M (2017). Nurse-led navigation to provide early palliative care in rural areas: a pilot study. BMC Palliat Care.

[28] Pawson R, Tilley N (1997). Realist evaluation.

[29] DeLurio J, Erinoff E, Hulshizer R (2015). Horizon scanning protocol and Operations Manual September 2015 Revision.

[30] Health Council of Canada. Innovation Practices Evaluation Framework. Available at: https://healthcouncilcanada.ca/470/, 2012.

[31] Valaitis RK, Carter N, Lam A (2017). Implementation and maintenance of patient navigation programs linking primary care with community-based health and social services: a scoping literature review. BMC Health Serv Res.

[32] Touzel M, Shadd J (2018). Content validity of a conceptual model of a Palliative Approach. J Palliat Med.

[33] Braun V, Clarke V et al. Thematic analysis. In: Cooper H, Camic PM, Long DL, eds. APA Handbook of Research Methods in Psychology, Research Designs. Washington: American Psychological Association 2012:57–71.

[34] Dalkin SM, Greenhalgh J, Jones D (2015). What’s in a mechanism? Development of a key concept in realist evaluation. Implement Sci.

[35] Wye L, Lasseter G, Percival J (2014). What works in ‘real life’ to facilitate home deaths and fewer hospital admissions for those at end of life? Results from a realist evaluation of new palliative care services in two English counties. BMC Palliat Care.

[36] Malcolm C, Knighting K (2022). A realist evaluation of a home-based end of life care service for children and families: what works, for whom, how, in what circumstances and why?. BMC Palliat Care.

[37] Fleming P, Sinnot M (2018). Rural physician supply and retention: factors in the Canadian context. Can J Rural Med.

[38] Rosser WW, Kasperski J (1999). Organizing primary care for an integrated system. Healthc Pap.

[39] Mangin D, Premji K, Bayoumi I et al. Brief on Primary Care Part 2: Factors Affecting Primary Care Capacity in Ontario for Pandemic Response and Recovery. Science Briefs of the Ontario COVID-19 Science Advisory Table. Toronto, Ontario, 2022.

[40] Boeckxstaens P, De Graaf P (2011). Primary care and care for older persons: position paper of the European Forum for Primary Care. Qual Prim Care.

[41] Elliott J, Stolee P, Boscart V (2018). Coordinating care for older adults in primary care settings: understanding the current context. BMC Fam Pract.

[42] Banfield M, Gardner K, McRae I (2013). Unlocking information for coordination of care in Australia: a qualitative study of information continuity in four primary health care models. BMC Fam Pract.

[43] Sweetman A, Buckley G. Ontario’s experiment with primary care reform. SPP Res Papers. 2014;7(11). 10.2139/ssrn.2434658.

[44] Valaitis R, Cleghorn L, Ploeg J (2020). Disconnected relationships between primary care and community-based health and social services and system navigation for older adults: a qualitative descriptive study. BMC Fam Pract.

[45] Rogers A, Flowers J, Pencheon D (1999). Improving access needs a whole systems approach. And will be important in averting crises in the millennium winter. BMJ.

[46] Nancarrow SA, Borthwick AM (2005). Dynamic professional boundaries in the healthcare workforce. Sociol Health Illn.

[47] Pearce C, Phillips C, Hall S (2011). Following the funding trail: financing, nurses and teamwork in Australian general practice. BMC Health Serv Res.

