# Supplementary material for: How do navigation programs address the needs of those living in the community with advanced, life-limiting Illness? A realist evaluation of programs in Canada
Source: BMC Palliat Care. 2023 Nov 15;22:179. doi: 10.1186/s12904-023-01304-3 (PMC10647106; doi:10.1186/s12904-023-01304-3)
Supplement: Supplementary file 1 — Supplementary Material 1 [file 12904_2023_1304_MOESM1_ESM.docx]

**Draft Interview Guide – Stakeholder / Decision / Policy Makers**

Questions for stakeholders who are involved in program delivery or management.

**About the program**

1. Please describe your role with respect to program X? How long have you been in this role?
2. Could you please describe the program?

Probes:

- - Who started the program?
  - What was the start date of the program?
  - What is the level of implementation? (Local, provincial, national?)
  - What is the target population?
  - Why was the program started?
  - What does it “look like”/how is it organized?
  - Where is it physically situated/located?
  - What is the governance structure?
  - What services are provided by the program?
  - Who are the key personnel involved in program delivery?

**Impacts of the program**

3. In what ways does the program address the needs of individuals affected by life-limiting illness and their families as they approach end-of-life (EOL)? In other words, what are the impacts of the program for patients and family/friend caregivers?

Probes:

- How does it improve awareness of available supports for end-of-life care?
- How does it connect patients and family/friend caregivers with services or supports?
- How does it improve coordination of care?

4. In your opinion, do you think the program has been successful in meeting the needs of individuals affected by life-limiting illness and their families who are living in the community? If so, how?

1. What aspects of the program contribute to the success described in #4?
2. [**Added in later interviews to refine emerging theory, as per realist methodology**] In the interviews we have done with other programs so far, there a few things that have come up consistently that have identified as contributing to the program’s success. Could you please comment on whether and how these things apply in relation to your program?

- Hiring the right people with the right skillsets
- Using a team approach
- The reputation of your program or organization
- Having the flexibility to respond to patient needs
- Program promotion and messaging

**Implementation and sustainability**

1. Were you involved in/do you have knowledge of the implementation process? If so, what were the barriers and facilitators to implementing the program? For example:

- Barriers associated with providers (e.g., collaboration problems, skills, trust), organizations (e.g., lack of support by leaders), health systems (e.g., resources, insufficient funds), and the program itself (e.g., requires complex actions to implement, perceived as adding questionable benefit).
- Facilitators or supports that helped with implementation.

1. Looking back, are there things that could have been done differently to improve the effectiveness of the program? Conversely, are there things that would be done the same way?
2. What are the resource and cost implications of the program? Consider the different inputs such as money investment, professional resources (e.g., number of persons, their profession, training, full- or part-time), technology, and time.
3. How do you see this program in the long-term?

Probes:

- - What are the long-term program objectives?
  - Do these differ from the initial program objectives?
  - Are there planned program changes on the horizon? If so, what do you hope these changes achieve?

1. What things would need to be put in place/remain in place in order for the program to continue to be successful?
2. Has an evaluation of the program been done? If so, what were the results? Please provide details regarding any evaluation, formal (e.g., program evaluation) or informal (e.g., short survey, anecdotes). Consider:
   - Process outcomes
   - Patient and family centered outcomes
   - Clinical outcomes
   - Overall implementation of the program

**Integration/interaction with health system and community**

1. How does the program interact with other aspects of the health care system (e.g., primary care, long-term care, home care, palliative care)?
2. Are there established processes/policies to communicate/coordinate with other aspects of the health care system?
3. Do program staff have established relationships with community-based health/social care programs and services? If so, please describe.
4. How does the program work with other aspects of the health care system and community-based programs and services to meet the needs of individuals affected by life-limiting illness and their families as they approach EOL?
